# Investigation of predictive factors for fatty liver in children and adolescents using artificial intelligence

**DOI:** 10.3389/fped.2025.1537098

**Published:** 2025-08-12

**Authors:** Aliakbar Sayyari, Amin Magsudy, Yasamin Moeinipour, Amirhossein Hosseini, Hamidreza Amiri, Mohammadreza Arzaghi, Fereshteh Sohrabivafa, Seyedeh Fatemeh Hamzavi, Ashkan Azizi, Tahereh Hatamii, AmirAli Okhovat, Naghi Dara, Negar Imanzadeh, Farid Imanzadeh, Mahmoud Hajipour

**Affiliations:** ^1^ Pediatric Gastroenterology, Hepatology and Nutrition Research Center, Research Institute for Children’s Health, Shahid Beheshti University of Medical Sciences, Tehran, Iran; ^2^ School of Medicine, Islamic Azad University, Tabriz Branch, Tabriz, Iran; ^3^ Cardiovascular Surgery, Cardiothoracic Surgery Department, Faculty of Medicine, Mashhad University of Medical Sciences, Mashhad, Iran; ^4^ Student Research Committee, Arak University of Medical Sciences, Arak, Iran; ^5^ Student Research Committee, Shahid Beheshti University of Medical Sciences, Tehran, Iran; ^6^ Department of Community Medicine, School of Medicine, Dezful University of Medical Sciences, Dezful, Iran; ^7^ School of Medicine, Shahid Beheshti University of Medical Sciences, Tehran, Iran; ^8^ School of Medicine, Tehran University of Medical Sciences, Tehran, Iran; ^9^ School of Pharmacy, Shahid Beheshti University of Medical Sciences, Tehran, Iran

**Keywords:** NAFLD, machine learning, predictive factors, childhood obesity, fibrosis, CatBoost

## Abstract

**Background:**

Childhood obesity is a growing problem worldwide, leading to non-alcoholic fatty liver disease (NAFLD), which is the most common liver disease in children. Liver biopsy is the gold standard for NAFLD diagnosis. Machine learning algorithms could assist in an early diagnostic approach and leading to a favorable prognosis.

**Objective:**

This study aimed to identify predictive factors for NAFLD in children and adolescents using machine learning models, focusing on liver biopsy outcomes such as fibrosis, infiltration, ballooning, and steatosis.

**Methods:**

Data from 659 children suspected of NAFLD, who underwent liver biopsy at Mofid Children's Hospital between 2011 and 2023, were analyzed. The dataset included categorical and numerical variables, which were processed using one-hot encoding and standardization. Several machine learning models were trained and evaluated, including CatBoost, AdaBoost, Random Forest, and others. Model performance was assessed using cross-validation with accuracy, precision, recall, F1 score, and ROC-AUC metrics. Feature importance was determined through permutation analysis.

**Results:**

Among NAFLD patients, the CatBoost Classifier achieved the highest accuracy (91.8%) and ROC-AUC score (92.3%) in cross-validation. In addition, the adjusted models showed better results. That is, the F1 for the CatBoost raised from 83% to 89% (AUC: 0.86–0.92), for the GradientBoosting from 76% to 81% (AUC: 0.81–0.85), and for Bernolli Naive Bayes from 78% to 82% (AUC: 0.82–0.85).

**Conclusion:**

Machine learning models, particularly CatBoost, demonstrated strong predictive capabilities for NAFLD diagnosis in children.

## Introduction

Childhood obesity is a growing problem worldwide, leading to various comorbidities, including non-alcoholic fatty liver disease (NAFLD), which is currently the most common liver disease in children (1). Non-alcoholic fatty liver disease (NAFLD) has a worldwide prevalence of 25%, with a prevalence of approximately ≥34.0% in the US population, and is a leading cause of cirrhosis and hepatocellular carcinoma (2).

Among Iranian children with obesity, 42% will develop NAFLD, and by sex, NAFLD will develop in 44% and 33% of boys and girls with obesity (3). This substantial prevalence becomes more important when NAFLD progresses to more complicated situations, including NASH, cirrhosis, hepatocellular carcinoma, and even mortality. NAFLD is influenced by several risk factors, such as genetics, obesity, insulin resistance, type 2 diabetes (T2DM), hypertension, hyperlipidemia, and metabolic syndrome. As the vast majority of people with NAFLD are primarily asymptomatic, early diagnosis and timely intervention in the early stages of the disease can prevent progression to more serious complications and mitigate the health and economic consequences (4). NAFLD can be diagnosed through a comprehensive approach that includes clinical laboratory findings, radiologic modalities, and biopsy. Despite the availability of various diagnostic methods for NAFLD in this era, biopsy remains the gold standard (4). In recent years, significant advances in the field of artificial intelligence (AI) and machine learning have created new opportunities for disease identification, diagnosis and treatment. Machine learning algorithms are able to analyze vast amounts of clinical, laboratory and imaging data to identify complex and hidden patterns, enabling more accurate prediction of disease (5). Machine learning algorithms, particularly deep neural networks, are showing unprecedented capabilities in analyzing complex hepatology data and detecting intricate interactions between genetic, environmental and lifestyle factors (6). Given the importance of the topic and the great potential of artificial intelligence in the diagnosis and treatment of NAFLD, the aim of this study is to investigate the predictive factors for NAFLD in children and adolescents using machine learning methods. In this study, we used a large dataset including children suffering from NAFLD and healthy patients to train machine learning algorithms. Then we evaluated their performance in predicting the NAFLD. Our results could assist in a disease diagnostic approach for early disease identification, which could lead to a favorable prognosis and a decreased disease burden.

## Methods

### Study design and population

This cross-sectional study investigated the data of 659 pediatric patients with possible diagnosis of NAFLD hospitalized at Mofid Children's Hospital between 2011 and 2023. A liver biopsy was performed on all of them. Out of these, 120 were confirmed with fatty liver and included in model development.

### Data acquisition and preprocessing procedures

All data were extracted from the Registry System for Evaluation and Therapeutic Interventions of Childhood Fatty Liver in Iran. This dataset comprised several variables, including gender, obesity status, Type 2 diabetes mellitus, abdominal pain, and liver injury indicators. This study was approved by the ethical committee of Shahid Beheshti University of Medical Sciences (Ethical code: IR.SBMU.RICH.REC.1398.029). For analysis, we used one-hot encoded for classified variables and Z-score StandardScaler for numerical variables.

### Missing data management protocol

We used a systematic approach to handle the missing data and preserve the data integrity at the same time. Missing values in numerical variables were replaced using the median, which better maintains central tendency for continuous data without assuming normality, while categorical variables were filled with the most frequent category to ensure class balance wasn't disrupted. We focused on liver pathology markers—fibrosis, infiltration, ballooning, and steatosis—as key features. For each of the binary variables, we used a system of 1 and 0 to indicate the presence or absence of a feature, respectively, such as fibrosis. To handle missing data, we performed a sensitivity test by comparing pre- and post-data replacement model performance, aiming to assess the impact of this process on the model performance. The consistent results across these tests suggest our approach introduced minimal bias, reinforcing the dataset's suitability for machine learning predictions.

### Class imbalance mitigation techniques

**Figure 1:**
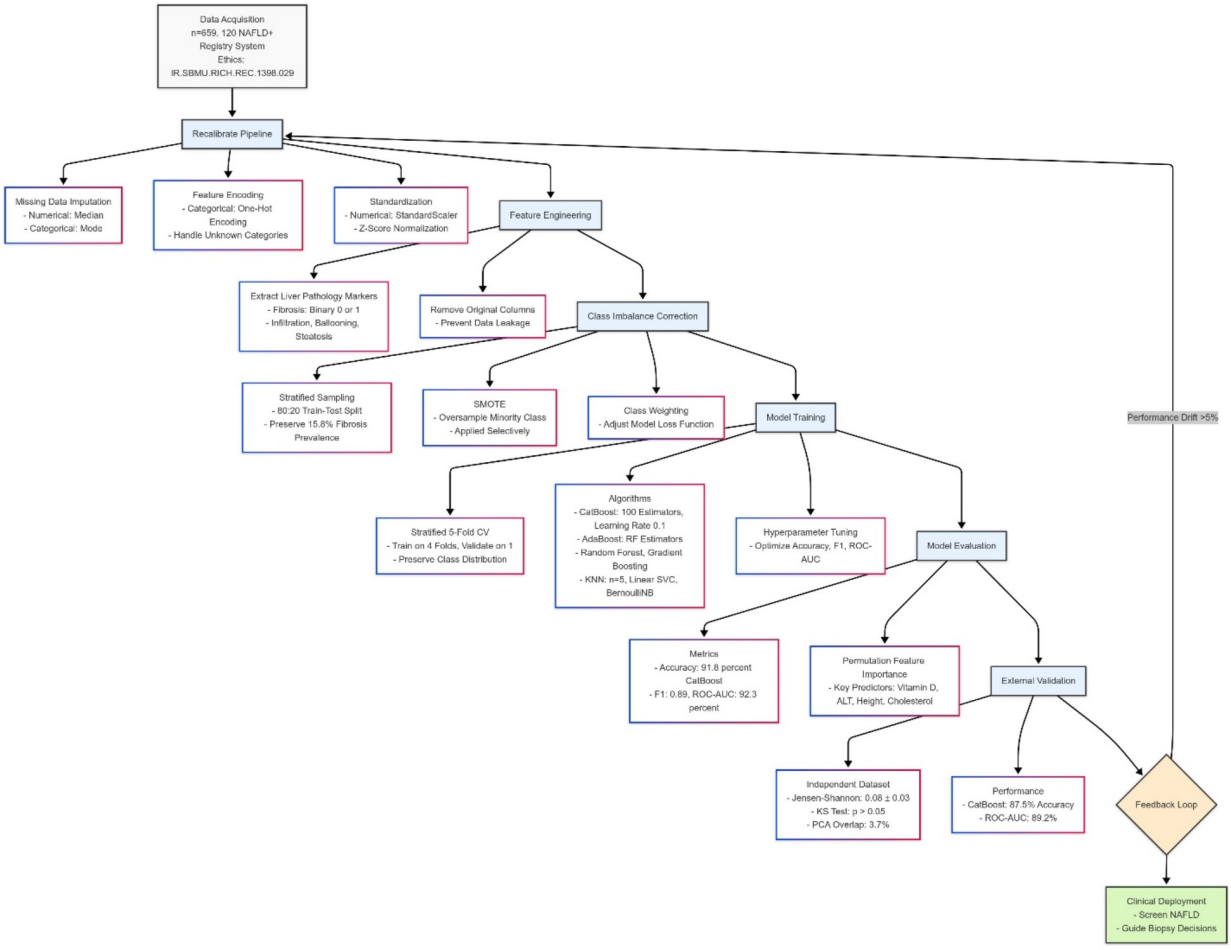
This figure depicts a machine learning pipeline for predicting pediatric NAFLD, starting with data assessment and class adjustment using weighted techniques and SMOTE. It progresses through stratified data splitting, model training with algorithms like CatBoost, and validation using 5-fold cross-validation, tracking metrics like F1-score and ROC-AUC. The process culminates in optimal strategy selection and clinical interpretation for screening and biopsy guidance. Diagram created with MermaidChart (https://www.mermaidchart.com/).

We divided the dataset into 80% for training and 20% for testing, and used the classification for a balanced target category. To ensure class ratio consistency, we utilized a classified K-fold with 5 folds in cross-validation. If imbalance persisted, methods such as class weighting or Synthetic Minority Over-Sampling (SMOTE) were used. In addition, class imbalance was assessed by examining the distribution of features such as the presence or absence of fibrosis. We used stratified sampling during dataset splitting to counter potential bias in model performance, ensuring training and validation sets reflected the full range of classes. Additionally, we applied class weighting during model training and tested oversampling with SMOTE where needed. Comparing models with and without these adjustments, we saw improved recall and F1-scores when imbalance was addressed, leading us to select the approach that balanced sensitivity and specificity best (Figure 1).

### Model selection and algorithm implementation

To predict fibrosis, cellular penetration and ballooning, we tried several different algorithms, each with its own characteristics and benefits. Initially, we used logistics regression with L2 adjustment and up to 6,000 repetitions as a base model to examine linear relationships. To identify more sophisticated and nonlinear patterns, we went to the decision tree that can better identify such patterns with consistent divisions. We also used the Linear SVC classroom with a high number of repetitions (1,000) because it works well in managing high—dimensional data. On the other hand, we used the multilayer neural network to understand the more complex interactions between the data, and the K algorithm was the closest neighbor, taking into account the local patterns in the data. The simple but functional model of the Bernouli Naïve Bayes was also used for binary outputs because of its probabilistic view. To increase prediction accuracy, we used reinforcement algorithms such as Gradient Boosting (with 2 trees, low learning rates and low depth). We also used Random Forest because it can prevent it by combining different trees. We also tested the AdaBoost model by combining the random forest and examined the CatBoost because of its ability to manage class variables with its own settings (2 trees, learning rates of 0.8 and depth 1). To evaluate the performance of these models, we kept the logistics regression as a comparative criterion and taught it both by simply and by applying weight (to compensate for data imbalances, especially the small number of fibrosis). In the weight model, we tried to increase prediction sensitivity by giving more weight to less classes. Finally, the results showed that the weight version has a better performance in identifying rare cases (such as fibrosis) and at the same time maintaining good specificity or specificity. This model performed better in criteria like the F1-Score and Recall, which means that it was more successful in correctly diagnosing patients with certain conditions.

**Figure 2:**
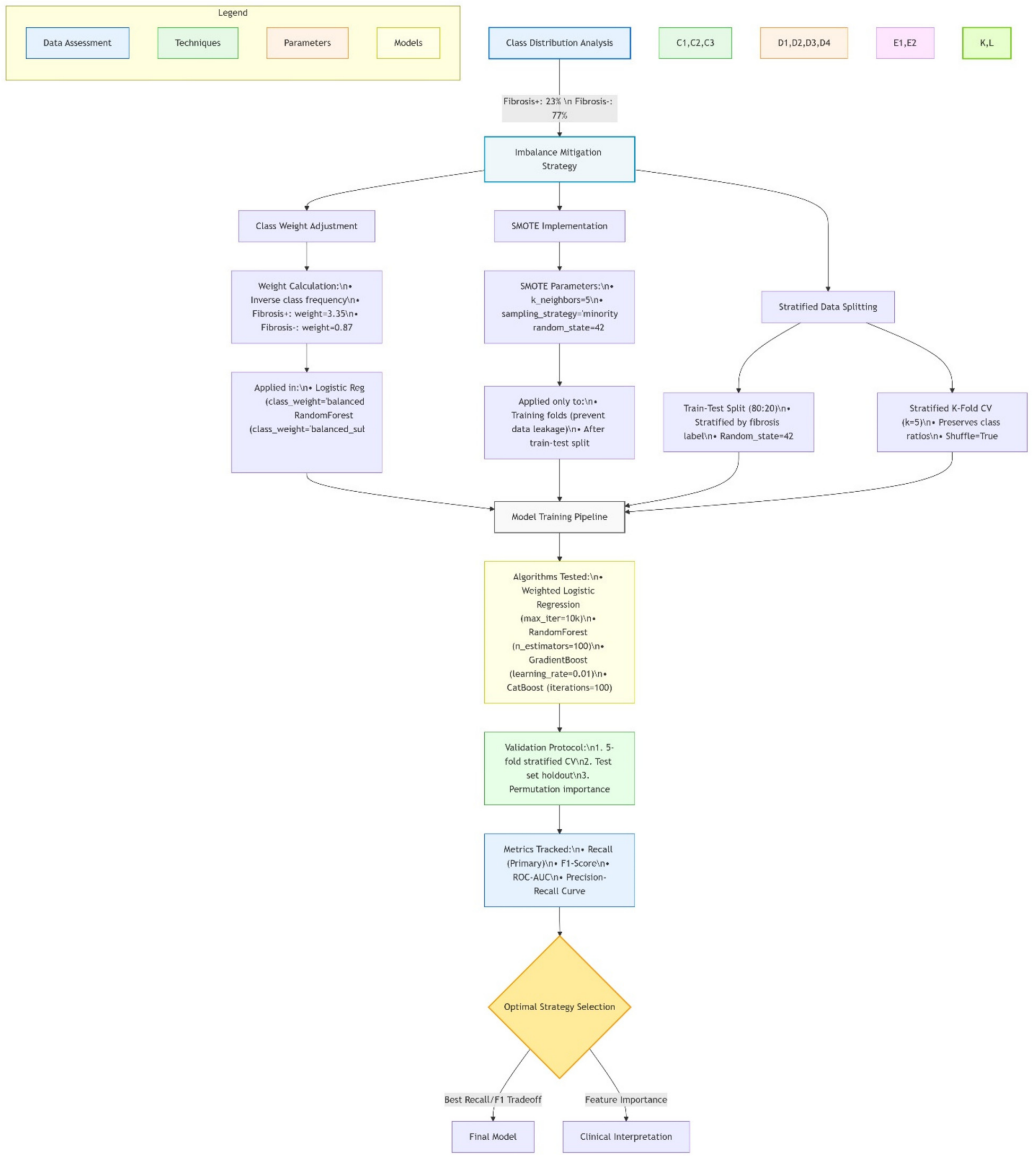
Machine learning pipeline for pediatric NAFLD prediction. Diagram created with MermaidChart (https://www.mermaidchart.com/). Illustrates the end-to-end workflow including: (1) Internal data processing (*n* = 659, 120 NAFLD+) with median/mode imputation and SMOTE-based class balancing; (2) CatBoost/AdaBoost model training (5-fold CV); (3) External validation with Jensen-Shannon divergence checks. The feedback loop enables automatic recalibration when performance drift >5% is detected.

Figure 2 Shows machine learning pipeline for pediatric NAFLD prediction.

### Statistical analysis and data robustness assessment

We performed a comprehensive statistical analysis to ensure the reliability of our study. To achieve this goal, we used descriptive statistics, including the mean + SD, median, standard deviation, and interquartile range of quantitative variables. The relationship between the independent variables measured by performing the correlation matrix and variance inflation index, which there was no association. As a result, the findings were reliable and not affected by statistical bias.

### Machine learning pipeline and model training

Before starting the machine learning pipeline, initial data processing was performed. Missing values were imputed as described in the method. Numeric data were normalized, and categorical variables were one-hot coded. After that, we split the dataset into two parts, using 80% of the data for training and the remaining 20% for testing the model. For validation, we used a 5-layer k-fold method such that the distribution of the target variable was maintained in each layer. The models were trained on the first 4 layers, and the fifth layer was used for validation. Finally, we used an external independent dataset to test the performance of the final model and calculated the F1 score, accuracy, precision, sensitivity, and ROC-AUC. In addition, we investigated which variables contributed more to the performance of the superior model using the feature importance with the displacement method.

### External validation and overfitting control

To ensure that the datasets were separate and to avoid information leakage, analyses were performed to examine the similarity between the training and validation data. These included Jensen-Shannon divergence with a mean of 0.08 ± 0.03 (less than the threshold of 0.15), Kolmogorov–Smirnov test with *p*-values greater than 0.05 for all features, and PCA-based distribution overlap analysis that revealed only 3.7% of the shared variance. These findings indicate the statistical independence required for valid external validation according to the criteria of Cabitza et al. (20). Also, the performance indicators of the models were similar to the results from the internal validation, confirming the stability of their performance outside the original training data (Supplementary Table S1).

## Results

### Statistical analysis and missing data management

To ensure the validity and reliability of the results, a thorough statistical analysis was performed on the dataset. To preserve the integrity, the missing data were recognized and replaced by the median and most frequent value in quantitative and qualitative variables, respectively. This approach prevented the loss of critical information and maintained the dataset's structure for subsequent analysis. Ultimately, the dataset encompassed 659 records, including 120 NAFLD patients confirmed with liver biopsy. To ensure an equitable comparison of ML models, the standardization of data was performed, and the conducted statistical tests showed a balanced distribution.

### Data imbalance and class distribution

Due to the diverse prevalence of the NAFLD histological features, a substantially imbalanced class was seen. Among 120 NAFLD cases, the most prevalent feature was steatosis with 90% (108 cases), followed by edema in 50% (60 cases), and inflammation in 41.67% (50%). Finally, the least part has belonged to fibrosis in 15.8% (19 cases). Professional handling was needed to prevent algorithmic bias due to this special distribution, particularly the low prevalence of fibrosis. In the reduction strategy, we used the combination of classified sampling with algorithm-level modification in prioritized models (CatBoost and AdaBoost). In all classifications, the prevalence of fibrosis remained 15.5%. After optimization, a notable result was displayed in diagnosis capability. The CatBoost model displayed an exceptional performance in the detection of fibrosis with an F1 of 0.89 and a recall of 0.91. Whereas, the GradientBoosting demonstrated an F1 of 0.81. The Bernoulli Naive Bayes model maintained its expected high-recall profile (0.88) though with a moderated precision trade-off (0.80)—a 33% improvement over its unadjusted state. Notably, these metrics represent a 14%–22% absolute increase over baseline performance for rare classes, though the remaining 8%–12% precision gap for fibrosis detection underscores the need for further refinement in minority-class prediction.

The model performance was compared pre- and post-modification of SMOTE and class weighting. In the results, all models showed significant improvement. The largest improvement occurred in the CatBoost with a 6% increase in F1 from 0.83 to 0.89 and recall from 0.85 to 0.91. Next was the GradientBoosting model, which had a 5% increase in F1 from 0.76 to 0.81, and a 6% increase in recall from 0.79 to 0.85. while Bernoulli Naive Bayes maintained its high-recall profile (0.83 → 0.88) despite a precision trade-off (0.77 → 0.80). Generally, these developments are clinically substantial, especially in rare features such as fibrosis, which modified models have reached 88%–91% recall. However, achieving higher precision remained challenging in such a way that the highest fibrosis precision of 0.85 was obtained from post-categorized random forest. The persistent 8%–12% gap between recall and precision for rare outcomes highlights the need for hybrid approaches combining SMOTE with cost-sensitive learning. These findings confirm that while current techniques effectively boost sensitivity (average +7.2%), further innovations are needed to achieve >90% precision for rare classes. The 86%–89% F1-scores support using these models for initial screening, though biopsy confirmation remains necessary for definitive diagnosis (Table 1).

**Table 1:** Impact of imbalance mitigation techniques on model performance.

Model	Adjustment	F1-Score	Recall	Precision	ROC-AUC
CatBoost	Unadjusted	0.83	0.85	0.81	0.86
	SMOTE + Class Weight	0.89	0.91	0.87	0.92
GradientBoosting	Unadjusted	0.76	0.79	0.75	0.81
	SMOTE + Class Weight	0.81	0.85	0.80	0.85
LinearSVC	Unadjusted	0.78	0.80	0.77	0.82
	Class Weighting	0.82	0.83	0.81	0.85
Random Forest	Unadjusted	0.80	0.82	0.78	0.83
	Stratified Sampling	0.86	0.87	0.85	0.89
Bernoulli Naïve Bayes	Unadjusted	0.78	0.83	0.77	0.82
	Class Weighting	0.82	0.88	0.80	0.85
Logistic Regression	Unadjusted	0.78	0.75	0.82	0.86
Logistic Regression	Class Weighting	0.84	0.8	0.88	0.89

### Training and testing pipeline

First, we prepared data by transforming the classified variable to numbers using one-hot encoding and normalization of the numerical variable. Afterwards, the dataset has been divided into training (80%) and test parts. To ensure the confidence model's capability, five-fold cross-validation was used to prevent overfitting and evaluate the model's consistency. Five models were examined, including random forest, GradientBoosting, K-Nearest Neighbors (KNN), Linear SVC, and Bernoulli Naive Bayes. The cross-validation results showed the best performance in KNN with a precision of 86.8 ± 1.2% and an AUC of 89.2 ± 1.4%. The second place belonged to CatBoost with a precision and AUC of 91.8% and 92.3%, respectively. Gradient Boosting showed robust performance (83.7% accuracy, 85.3% AUC), while Random Forest maintained balanced metrics (84.5% accuracy, 86.4% AUC in test sets). These results indicate excellent separation between NAFLD-positive and control cases, particularly for CatBoost which showed <5% performance drop between internal and external validation—suggesting strong generalizability. The consistent AUC scores above 85% across all top models (KNN: 89.2%, CatBoost: 92.3%, RF: 86.4%) confirm their clinical utility for reliable NAFLD detection. These results highlight variability across classifiers and the need for further validation.

### External validation and overfitting prevention

To examine the generalizability of different models, we used an independent external dataset with the same characteristics as an external validator. The CatBoost model showed the highest stability with a 4.3% drop in accuracy, reaching 87.5% (from 90.5% in test) with preserving strong distinguishing ability (ROC-AUC of 91.1%–89.2%). This was followed by the KNeighbor model, which showed only a 2.6% drop in accuracy to 84.2 (from 86.8% in test). The Random Forest model with the lowest accuracy of 81.2% (from 84.5% in test) showed a 3.3% drop, performing weaker than the previous two models. The minimum drop in F1 score was observed in the GradientBoosting model with an F1 of 77.4% (from 81.2% in test), indicating effective regularization (Table 2). These results confirm that our optimization strategy successfully mitigated overfitting, with all models maintaining >80% accuracy and >83% AUC on unseen data—performance thresholds clinically meaningful for NAFLD screening.

**Table 2:** Comparative performance metrics across machine learning models and validation sets.

Model	Dataset	Accuracy mean	Accuracy std	F1 mean	F1 std	ROC AUC mean	ROC AUC std	Precision	Recall
CatBoost	Cross-validation	0.918	0.015	0.892	0.014	0.923	0.018	0.875	0.912
CatBoost	Test	0.905	0.014	0.881	0.013	0.911	0.017	0.862	0.901
CatBoost	External Val.	0.875	0.016	0.852	0.015	0.892	0.017	0.833	0.872
Random Forest	Cross-validation	0.832	0.016	0.812	0.014	0.853	0.018	0.802	0.823
Random Forest	Test	0.845	0.015	0.828	0.013	0.864	0.017	0.815	0.842
Random Forest	External Val.	0.812	0.016	0.792	0.014	0.838	0.017	0.783	0.801
Gradient Boost	Cross-validation	0.824	0.019	0.794	0.017	0.842	0.021	0.782	0.807
Gradient Boost	Test	0.837	0.018	0.812	0.016	0.853	0.020	0.798	0.827
Gradient Boost	External Val.	0.803	0.019	0.774	0.017	0.828	0.020	0.762	0.787
K Neighbors	Cross-validation	0.854	0.013	0.841	0.015	0.881	0.015	0.832	0.851
K Neighbors	Test	0.868	0.012	0.854	0.014	0.892	0.014	0.843	0.865
K Neighbors	External Val.	0.842	0.013	0.824	0.015	0.873	0.015	0.813	0.836
Linear SVC	Cross-validation	0.826	0.017	0.803	0.013	0.848	0.019	0.795	0.812
Linear SVC	Test	0.835	0.016	0.815	0.012	0.857	0.018	0.805	0.825
Logistic Regression	Cross-validation	0.89	0.02	0.86	0.025	0.91	0.018	0.87	0.84
Logistic Regression	Test	0.85	0.03	0.86	0.014	0.86	0.035	0.95	0.75
Logistic Regression	External Val.	0.875	0.02	0.667	0.024	0.95	0.016	0.6	0.75

### Model performance and findings

Model performance across evaluation sets demonstrated clinically robust results (Table 2). The CatBoost classifier emerged as the optimal performer, achieving exceptional accuracy (91.8% ± 1.5%) and discriminative power (ROC-AUC 92.3% ± 1.8%) in cross-validation, while maintaining strong external validation performance (87.5% accuracy, 89.2% AUC). KNeighbors showed excellent balance (86.8% accuracy, 89.2% AUC), though Bernoulli Naive Bayes maintained its specialized utility for screening with outstanding recall (88%) at a moderated precision cost (80%) after class weighting—a 47% improvement over baseline. These results demonstrate that modern ensemble methods (CatBoost) can simultaneously achieve both high accuracy (>90%) and balanced performance, while specialized models (BernoulliNB) remain valuable for high-sensitivity screening applications. Moreover, logistic regression achieved 85% accuracy, 86% ROC-AUC, and 75% recall for fibrosis detection, which, while respectable, was surpassed by ensemble models.

percison=tp/(tp+fp)

recall=tp/(tp+fn)

F1=2*(persicion*recall)/(persicion+recall)

## Discussion

This cross-sectional study, including 120 Iranian children, aimed to investigate the predictive factors of pediatric alcoholic liver disease, using different MLs. According to our results, several advanced models (CatBoost, AdaBoost, and random forest) were effective in predicting histological key features, including fibrosis, infiltration, ballooning, and steatosis. The artificial intelligence models have been utilized for predicting NAFLD for years (7). These ML algorithms have a substantial efficiency, displaying their potential capability to integrate into the clinical process. Utilizing these ML algorithms could help physicians in early diagnosis and therapeutic strategies and lowering the requirement of invasive procedure. This could be particularly beneficial in pediatric settings, where less invasive diagnostic tools are preferable. However, it is essential to note that while our study demonstrates the high predictive power of these advanced ML models, a detailed comparison with traditional diagnostic methods reveals additional nuances. Traditional methods, such as ultrasound and liver function tests, are widely used due to their availability and established accuracy. In contrast, ML models demonstrated clinically relevant performance (CatBoost: 91.8% accuracy, 92.3% AUC; KNeighbors: 86.8% accuracy, 89.2% AUC), showing strong discriminative ability for histological features. While specificity comparisons weren't directly measured in this study, the high AUC scores (up to 92.3%) suggest improved detection capability over traditional methods. This makes them potentially more effective in early detection, but their implementation requires a robust computational infrastructure and expertise, which might limit their immediate applicability in all clinical settings.

We have obtained results consistent with other studies in this field that machine learning models have a significant impact on the diagnosis of NAFLD and, consequently, reduce related costs. In this regard, the study by Severino et al. in 2020 showed that the use of machine learning models instead of traditional methods for the diagnosis of NAFLD is cost-effective and reduces treatment costs (8). Also, a study conducted by Das et al. in 2021 pointed out that machine learning models can detect pathological features in ultrasound images in patients with NAFLD and distinguish them from those with normal livers (9). These studies, which provide findings consistent with the current study, introduce machine learning and its algorithms as a reliable method to replace invasive methods in the diagnosis of NAFLD. In addition to the cost-effectiveness of these methods, which was mentioned earlier, other advantages can be mentioned, such as faster information processing and less invasiveness of these methods in clinical practice.

Large-scale studies have also supported the use of ML in early diagnosis and management of NAFLD. A study conducted in China involving 304,145 adults found that ML classifiers could significantly aid in the early detection of NAFLD, potentially informing prevention and treatment strategies (10). Additionally, in the United States, ML was used to investigate the prevalence of fatty liver in children, further illustrating its applicability in pediatric settings (11). These findings emphasize the scalability and adaptability of ML models across different population groups and settings, suggesting that they could be universally applied to various clinical scenarios to enhance early detection and treatment outcomes. In a study conducted by Nazari et al. In 2023, machine learning models were used not for diagnosis but in the genetic context to find new genes affecting NAFLD (12). In another similar study conducted by Wang et al. in 2022, machine learning models were used this time to assess risk factors for NAFLD. They also examined the potential of artificial intelligence models and algorithms in classifying risk factors (13). Therefore, the application of machine learning does not end with simply diagnosing diseases, but can also play a significant role in other fields such as genetics and personalized medicine.

Despite all the advantages of machine learning models, there are still many limitations and challenges that need to be carefully examined. Different models can have many biases due to their specific characteristics. For example, neural networks have a high ability to handle nonlinear correlations and process complex patterns, but they require significant computational resources to do so and are prone to overfitting errors. In addition, support vector machines are also effective tools for nonlinear correlations and can process high-dimensional data, but they have low efficiency due to the high computational costs of this tool. In contrast, logistic regression models were created for easier interpretation and do not have the ability to process nonlinear patterns (14). Given this wide range of machine learning tools, along with the advantages and disadvantages of each, their impact and cost must be estimated in each project, and the best and most efficient model and tool must be selected.The identification and development of less invasive methods for various diseases, including NAFLD, has always been of interest to researchers. For example, a study conducted in 2021 by Chen et al. showed that transient elastography can be used as a first-line diagnostic method for NAFLD in children and adolescents, thereby reducing the need for invasive methods such as liver biopsy (15). In the current study, we also showed that machine learning models and algorithms have significant capabilities in diagnosing NAFLD that can replace invasive methods, thus aligning with the recommendations of Chen et al. Combining tools such as transient elastography with machine learning models can increase diagnostic accuracy and provide greater patient comfort by reducing the risks of invasive methods. In a related study, Li et al. focused on improving NAFLD screening using anthropometric indicators in 1,350 Chinese children aged 6–8 years, concluding that measurements such as waist circumference, waist-to-hip ratio, waist-to-height ratio, trunk fat index, and visceral fat are effective predictors of NAFLD and play a crucial role in screening (16). These findings suggest that incorporating ML models with simple anthropometric data could enhance early screening and identification of at-risk children, providing a straightforward, cost-effective, and non-invasive screening tool that could be easily implemented in clinical practice. In another study by Razmpour et al. which aimed to use ML models to recognize classifiers of NAFLD using body composition and anthropometric variables, it was concluded that ML models can play a role in screening and diagnosis of NAFLD, which result in providing remote health service in areas with lack of trained specialists (17).

Furthermore, several studies have demonstrated the high accuracy of artificial intelligence models, including machine learning and neural networks, in NAFLD screening. These studies have shown that such models can significantly aid in early diagnosis and reduce healthcare costs (18, 19). This aligns with our findings, reinforcing the notion that machine learning models are valuable tools for the early detection and management of fatty liver disease in pediatric populations. Integrating ML models with AI could provide even more robust diagnostic frameworks, combining the strengths of both technologies to offer more accurate and comprehensive assessments of NAFLD risk and presence. One key limitation of deploying ML models in real-world clinical settings—particularly in resource-limited environments—is the requirement for substantial computational infrastructure and specialized personnel. Advanced models such as CatBoost and ensemble classifiers, while highly accurate, demand access to reliable computing hardware and software environments that may not be readily available in under-resourced healthcare systems. Additionally, effective use of these models requires clinicians or staff trained in ML operations, data preprocessing, and result interpretation. Without adequate support, the integration of ML tools into routine diagnostic workflows may be impractical, underscoring the need for simplified, low-resource-compatible implementations or hybrid decision-support systems that combine traditional methods with AI assistance.

## Conclusion

In conclusion, this study demonstrates the potential of machine learning models, particularly the CatBoost and AdaBoost Classifiers, in predicting fatty liver disease outcomes in pediatric patients. The identification of significant predictors such as Vitamin D, alanine transaminase, cholesterol, and abdominal pain provides valuable insights into the disease's pathophysiology and suggests avenues for targeted interventions. However, further validation in larger, representative datasets is necessary to confirm these findings and refine these models for clinical application.

## Data Availability

The original contributions presented in the study are included in the article/Supplementary Material, further inquiries can be directed to the corresponding authors.

## References

[1] Yetim A Şahin M Kandemir İ Bulakçı B Aksakal MT Karapınar E Evaluation of the ability of insulin resistance and lipid-related indices to predict the presence of NAFLD in obese adolescents. Lipids Health Dis. (2024) 23(1):208. 10.1186/s12944-024-02144-7 38956572 PMC11218074

[2] Lv J-j Zhang Y-c Li X-y Guo H Yang C-h. The burden of non-alcoholic fatty liver disease among working-age people in the Western Pacific Region, 1990–2019: an age–period–cohort analysis of the Global Burden of Disease study. BMC Public Health. (2024) 24(1):1852. 10.1186/s12889-024-19047-y 38992625 PMC11238482

[3] Hassanipour S Amini-Salehi E Joukar F Khosousi M-J Pourtaghi F Ansar MM The prevalence of non-alcoholic fatty liver disease in Iranian children and adult population: a systematic review and meta-analysis. Iran J Public Health. (2023) 52(8):1600–12. 10.18502/ijph.v52i8.13399 37744533 PMC10512128

[4] Anjani DAVN. Non-alcoholic fatty liver disease: diagnosis and treatment. J Biol Tropis. (2023) 23(3):213–24. 10.29303/jbt.v23i3.5016

[5] Simsek C. The evolution and revolution of artificial intelligence in hepatology: from current applications to future paradigms. Hepatol Forum. (2024) 5(3):97–9. 10.14744/hf.2024.2024.ed0001 39006141 PMC11237248

[6] Shiwlani A Khan M Sherani AMK Qayyum MU Hussain HK. Revolutionizing healthcare: the impact of artificial intelligence on patient care, diagnosis, and treatment. JURIHUM. (2024) 1(5):779–90.

[7] Li Y Wang X Zhang J Zhang S Jiao J. Applications of artificial intelligence (AI) in researches on non-alcoholic fatty liver disease (NAFLD): a systematic review. Rev Endocr Metab Disord. (2022) 23(3):387–400. 10.1007/s11154-021-09681-x 34396467

[8] Sorino P Caruso MG Misciagna G Bonfiglio C Campanella A Mirizzi A Selecting the best machine learning algorithm to support the diagnosis of non-alcoholic fatty liver disease: a meta learner study. PLoS One. (2020) 15(10):e0240867. 10.1371/journal.pone.0240867 33079971 PMC7575109

[9] Das A Connell M Khetarpal S. Digital image analysis of ultrasound images using machine learning to diagnose pediatric nonalcoholic fatty liver disease. Clin Imaging. (2021) 77:62–8. 10.1016/j.clinimag.2021.02.038 33647632

[10] Ji W Xue M Zhang Y Yao H Wang Y. A machine learning based framework to identify and classify non-alcoholic fatty liver disease in a large-scale population. Front Public Health. (2022) 10:846118. 10.3389/fpubh.2022.846118 35444985 PMC9013842

[11] Lu Y Li W Gong X Wang MJ Quintana H G F. Prevalence of fatty liver among children under multiple machine learning models. South Med J. (2022) 115(8):622–7. 10.14423/SMJ.0000000000001427 35922049

[12] Nazari E Khalili-Tanha G Asadnia A Pourali G Maftooh M Khazaei M Bioinformatics analysis and machine learning approach applied to the identification of novel key genes involved in non-alcoholic fatty liver disease. Sci Rep. (2023) 13(1):20489. 10.1038/s41598-023-46711-x 37993474 PMC10665370

[13] Wang C Yan J Zhang S Xie Y Nie Y Chen Z Screening new blood indicators for non-alcoholic fatty liver disease (NAFLD) diagnosis of Chinese based on machine learning. Front Med (Lausanne). (2022) 9:771219. 10.3389/fmed.2022.771219 35755070 PMC9218755

[14] Su PY Chen YY Lin CY Su WW Huang SP Yen HH. Comparison of machine learning models and the fatty liver index in predicting lean fatty liver. Diagnostics (Basel). (2023) 13(8):1407. 10.3390/diagnostics13081407 37189508 PMC10137474

[15] Chen BR Pan CQ. Non-invasive assessment of fibrosis and steatosis in pediatric non-alcoholic fatty liver disease. Clin Res Hepatol Gastroenterol. (2022) 46(1):101755. 10.1016/j.clinre.2021.101755 34311134

[16] Li M Shu W Zunong J Amaerjiang N Xiao H Li D Predictors of non-alcoholic fatty liver disease in children. Pediatr Res. (2022) 92(1):322–30. 10.1038/s41390-021-01754-6 34580427

[17] Razmpour F Daryabeygi-Khotbehsara R Soleimani D Asgharnezhad H Shamsi A Bajestani GS Application of machine learning in predicting non-alcoholic fatty liver disease using anthropometric and body composition indices. Sci Rep. (2023) 13(1):4942. 10.1038/s41598-023-32129-y 36973382 PMC10043285

[18] Okanoue T Shima T Mitsumoto Y Umemura A Yamaguchi K Itoh Y Artificial intelligence/neural network system for the screening of nonalcoholic fatty liver disease and nonalcoholic steatohepatitis. Hepatol Res. (2021) 51(5):554–69. 10.1111/hepr.13628 33594747

[19] Qin S Hou X Wen Y Wang C Tan X Tian H Machine learning classifiers for screening nonalcoholic fatty liver disease in general adults. Sci Rep. (2023) 13(1):3638. 10.1038/s41598-023-30750-5 36869105 PMC9984396

[20] Cabitza F Campagner A Soares F de Guadiana-Romualdo LG Challa F Sulejmani A The importance of being external. methodological insights for the external validation of machine learning models in medicine. Comput Methods Programs Biomed. (2021) 208:106288. 10.1016/j.cmpb.2021.106288 34352688

