# Supplementary material for: Investigation of predictive factors for fatty liver in children and adolescents using artificial intelligence
Source: Front Pediatr. 2025 Aug 12;13:1537098. doi: 10.3389/fped.2025.1537098 (PMC12377039; doi:10.3389/fped.2025.1537098)
Supplement: Supplementary file 1 [file Table1.docx]

**Supplementary table 1: Similarity Assessment Metrics Between Training and Validation Datasets.**

| **Metric** | **Value** | **Threshold** | **Interpretation** |
| --- | --- | --- | --- |
| Jensen-Shannon Divergence | 0.08 | <0.15 | No significant distribution shift |
| KS Test p-values (mean) | 0.21 | >0.05 | Feature distributions match |
| PCA Shared Variance | 3.7% | <5% | Minimal dataset overlap |
